# Pulsatile Hyperglycaemia Induces Vascular Oxidative Stress and GLUT 1 Expression More Potently than Sustained Hyperglycaemia in Rats on High Fat Diet

**DOI:** 10.1371/journal.pone.0147412

**Published:** 2016-01-20

**Authors:** Günaj Rakipovski, Jens Lykkesfeldt, Kirsten Raun

**Affiliations:** 1 Department of Experimental Animal Models, Section of Veterinary Disease Biology, Faculty of Health and Medical Sciences, University of Copenhagen, Copenhagen, Denmark; 2 Department of Diabetes Pharmacology, Global Research, Novo Nordisk A/S, Måløv, Denmark; Institut d'Investigacions Biomèdiques August Pi i Sunyer, SPAIN

## Abstract

**Introduction:**

Pulsatile hyperglycaemia resulting in oxidative stress may play an important role in the development of macrovascular complications. We investigated the effects of sustained vs. pulsatile hyperglycaemia in insulin resistant rats on markers of oxidative stress, enzyme expression and glucose metabolism in liver and aorta. We hypothesized that liver’s ability to regulate the glucose homeostasis under varying states of hyperglycaemia may indirectly affect oxidative stress status in aorta despite the amount of glucose challenged with.

**Methods:**

Animals were infused with sustained high (SHG), low (SLG), pulsatile (PLG) glucose or saline (VEH) for 96 h. Oxidative stress status and key regulators of glucose metabolism in liver and aorta were investigated.

**Results:**

Similar response in plasma lipid oxidation was observed in PLG as in SHG. Likewise, in aorta, PLG and SHG displayed increased expression of glucose transporter 1 (GLUT1), gp-91^PHOX^ and super oxide dismutase (SOD), while only the PLG group showed increased accumulation of oxidative stress and oxidised low density lipoprotein (oxLDL) in aorta.

**Conclusion:**

Pulsatile hyperglycaemia induced relatively higher levels of oxidative stress systemically and in aorta in particular than overt sustained hyperglycaemia thus supporting the clinical observations that pulsatile hyperglycaemia is an independent risk factor for diabetes related macrovascular complications.

## Introduction

Recent epidemiological studies and controlled intervention trials have shown that despite having similar average glycaemic exposure—as measured by glycosylated haemoglobin (HbA1c)—the risks of developing micro and macro-vascular complications can be different in diabetic subjects suggesting the need for renewed focus on other risk markers [1,2]. Postprandial hyperglycaemia that is typically not reflected in an altered HbA1c status has been hypothesized to be an interesting and independent risk factor for the incident of cardiovascular events in diabetic patients [2–4]. Several in vitro and in vivo studies both in humans and animals have reported that repeated episodes of hyperglyceamic “spikes” may induce higher levels oxidative stress than sustained hyperglycaemia and thus be more detrimental to the cardio vascular system [5–8]. However, the exact sequence of the biochemical and molecular events during postprandial hyperglycaemia are still not fully understood.

Recently, it has been reported that during chronic hyperglycaemia cells may metabolically adapt to situations of excess glucose and thus exert protective mechanisms against oxidative stress [9]. One putative mechanism may be the development of insulin resistance and the resulting down-regulation of glucose uptake [10]. In liver, glucose uptake and metabolism are strictly regulated by several enzymes such as the glucokinase (GK), glycogen synthase (GS) and the Sterol Regulatory Element-Binding Proteins (Srebp1c)[11]. The expression of liver GK, the rate limiting enzyme for glucose uptake, is primarily controlled by insulin and down regulation of liver GK has been shown both in insulin resistant and diabetic models[12,13]. Paradoxically, the protective down regulation of glucose uptake by insulin responsive tissues may leave vascular endothelial cells (VEC) as important targets of hyperglycaemia mediated oxidative insults[14]. Excess glucose influx in VEC by facilitated diffusion through the GLUT1[15] may activate protein kinase C (PKC) and consequently augment the activity of the NADPH oxidase enzyme complex (NOX) resulting in increased reactive oxygen species (ROS) and oxidative stress[16–18]. This in turn may increase the accumulation of oxidised low density lipoprotein (oxLDL) in vessels and consequently lead to plaque formation and atherosclerosis[19]. ROS molecules may be neutralized by the superoxide dismutase (SOD) but studies have shown that this defence mechanism may be depressed during diabetes thus leading to a further oxidative damage[20]. In the present study, we assessed the effects of sustained vs. pulsatile hyperglycaemia in high fat fed rats accompanied with insulin resistance on markers of oxidative stress, expression of key enzymes and glucose metabolism in liver and aorta. We hypothesized that regulation of glucose metabolism between insulin responsive, such as the liver and non-responsive tissues such as the vascular system in form of aorta may also be dependent on the hyperglycaemic profile rather and not only by the total glycaemic exposure. In turn this may indirectly impact the severity of oxidative stress at different tissue level.

## Materials and Methods

### Animals and surgery

The study was approved by the Danish Animal Experimentation Inspectorate under the Ministry of Justice and carried out by trained and licensed personnel. A total of 30 Sprague-Dawley male rats (Taconic, Denmark), were used in the study. Animals were high fat feed (D12492, Research Diets, USA) for at least 20 weeks in order to develop obesity, insulin resistance and dyslipidemia. Animals had free access to food and water (tap water added 1% citric acid) and housed in pairs with 12h light: 12h dark cycle in temperature (22 ± 2°C) and humidity (50± 20%) controlled rooms. Prior to surgery, animals were treated with Anorfin® (GEA A/S, Denmark) and subsequently kept on anaesthesia with Isofluran® (Baxter Pharmaceuticals, USA). Silicon catheters (Tygon Microbore Tubing, S-50-HL, Cole Parmer, UK.) were placed in jugular vein and in carotid artery. Catheters was externalized subcutaneously, to the mid-scapular region, filled with 500IU/ml heparin (LEO Pharma Nordic, Denmark) in HAEMACCEL^®^ (Intervet/Schering-plough, UK) and sealed into Dacron buttons (Instech Laboratory Inc, USA). After surgery animals were single housed and allowed 7–8 days of recovery and treated with Baytril® (Bayer AG, Germany) and Rimadyl® (Pfizer Inc, USA) for three days.

### Infusion protocol

Animals were randomized into four weight-matched groups (n = 7–8). Catheters were flushed with heparin solution (100 U/ml in saline; LEO Pharma Nordic, Denmark) to ensure proper function. Carotid artery catheters were connected to an Accusampler® (DiLab, Sweden) for automated blood sampling whereas the jugular vein catheters were connected to the infusion pump (World Precision Instruments Inc. USA). The animals were given intravenous infusion of glucose (50% glucose solution; Fresenius Kabi AG, Germany) or saline for 96 hours as outlined in Table 1.

**Table 1:** Infusions rate (ml/kg/h). The table describes one cycle of vehicle or glucose infusion and the amount of glucose infused per 24 hours. The cycle was repeated continuously during the complete infusion period of 96 hours.

	0–2 min	2–32 min	32–152 min	Glucose (g/kg/24 hours)
Saline (VEH)	1.0	1.0	1.0	0
Sustained High Glucose (SHG)	3.0	3.0	3.0	36.0
Sustained Low Glucose (SLG)	1.0	1.0	1.0	12.0
Pulsatile Glucose (PLG)	30.0	3.5	0	12.4

### Blood sampling

Blood samples were drawn by the accusampler system, in K_3_-EDTA coated tubes and centrifuged (4°C; 5000rpm; 3 min). Blood (20 μl) for glucose and insulin were drawn at 0.0 0.2 0.8 6.0 6.2 6.8 24.0 24.2 24.8 32.0 32.2 32.8 48.0 48.2 48.8 66.0 66.2 66.8 72.0 72.2 72.8 96.0 96.2 96.8 hours in order to cover the entire circadian rhythm and the various glucose profiles. Blood (100μl) for Plasma total cholesterol (T-Chol), low density lipoprotein (LDL), High density lipoprotein (HDL), triglycerides (TG), Glucagon, MDA and Plasma 8- isoprostanes (8-IsoP) analyses were drawn at time points 0, 24, 48, 72 and 96 hours.

### Study termination

After 96 hours of infusion, animals were disconnected from the accusampler system and infusion pumps and anesthetized with isofluran. The abdominal cavity was opened and liver and thoracic aorta was excised, rinsed, and preserved in liquid nitrogen. Subsequently and while still anaesthetized, animals were euthanized by a cardiac injection of pentobarbital (100μl; 200mg/ml).

### Biochemical analyses

For glucose measurement, blood (5 μl) were diluted in 500 μl of EBIO buffer solution (Eppendorf, Hamburg, Germany) and analysed by immobilized glucose oxidase methodology (EBIO Plus autoanalyzer; Eppendorf, Germany). Plasma insulin were analysed by rat insulin sensitive ELISA (Chrystal Chemistry, USA). T-Chol, LDL, HDL and TG were measured on a Hitachi 912 analyzer using commercial available assay kits (TG: Cat. No. 11488872; T-Chol: Cat. No. 1489232; HDL: Cat. No.3038661; LDL: Cat. No. 1985604 Roche diagnostics; Switzerland). Plasma Glucagon levels were measured by Rat glucagon sensitive ELISA (Cat. No. 48-GLUHU-E01; ALPCO, USA).Lipid oxidation was assessed by measuring MDA in plasma, liver and aorta as described previously [21]. Liver TG and glycogen (GLY) content were determined by homogenizing weighed liver tissue with a reagent consisting of a sodium acetate buffer mixed with Triton X-100 for 15 seconds by use of homogenizer, Polytron PT 3000 (PT-DA 3007/2 generator, IKA-Werke Germany). After homogenization, the sample were boiled for two minutes, and thereafter kept on slush ice for fast cooling. The homogenate was centrifuged and analysed for TG on a Hitachi 912 analyser (Cat. No. 11488872; Roche diagnostics; Switzerland). For glycogen analysis, 25 μl amyloglucosidase (Sigma-Aldrich) was added to the homogenate and incubated at 20°C overnight before of total glucose content. SOD in liver and aorta was assessed as described by manufacturer (Sigma Aldrich Cat. No. 19160). OxLDL in aorta was assessed as described manufacturer (Uscn Life Science Inc; China, Cat. No. E90527Ra).8-IsoP levels were measured as described by the assay kit manufacturer (Cat no.:516351, Cayman Chemicals, USA).

### Western Blotting

Tissue protein lysates were assayed with antibodies against Glycogen Synthase (Cell Signalling Technology, USA), GLUT1 and gp-91^PHOX^ (Abcam, USA) GK and Srebpc1 (2A4) (Santa Cruz, USA). Protein levels were normalized to β-Actin (Abcam, USA). Secondary antibodies were horseradish peroxidise-coupled and ECL reagent (BioVision, USA) was used for detection. Quantification was performed using ImageGuage 4.0 (Fujifilm, Japan).

### Real-time PCR

RNA was isolated from tissue using the RNeasy Mini Kit (Qiagen). cDNA was synthesized using the iScript kit (Bio-Rad). Primer-probesets were from TaqMan Gene Expression Assays (Applied Biosystems) (except Srebp1c) and PCR reactions were performed using a TaqMan Master mix (Applied Biosystems) and a MX3000P system (Agilent). Srebp1c custom primer-probe sets were purchased from Applied Biosystems with the sequences: Forward: CGCTACCGTTCCTCTATCAATGAC; Reverse: AGTTTCTGGTTGCTGTGCTGTAAG; Probe: GTGGTGGGCACTGAGGC.

Ct values were normalized to those of Ppib.

### Statistics

Statistical analyses of the results were performed by using SAS JMP software (version 8.1 for Windows, SAS institute, Cary, NC). Plasma MDA, T-Chol, LDL, HDL and TG were analysed using ANCOVA with time 0 hours as covariant and then a fit model with time, treatment and time x treatment interaction as model based variables. Liver and aorta metabolic parameters were analysed using ANOVA. In cases of statistical significance, Tukey’s post hoc test was applied. A p-level less than 0.05 was considered statistically significant.

## Results

### Pulsatile hyperglycaemia increased systemic oxidative stress

Baseline plasma glucose (5–6 mmol/l) and insulin (640 pmol/l) were not different between groups. In the SHG group, hyperglycaemia (>22 mmol/l) and hyperinsulinemia (>3000 pM) were immediately manifested and maintained throughout the study. The SLG group, receiving one third the amount of glucose of the SHG group, displayed a moderate increase in glycaemia (~12 mmol/l) at the initial phase of experimentation but levelled out during the time course. Interestingly, the plasma insulin levels for the SLG group were steadily increasing during the time course reaching to levels of approximately 2000 pmol/l. The PLG group, receiving the same amount of glucose as the SLG group but by pulsatile infusion, showed the expected fluctuations in both plasma glucose and insulin. The plasma glucose levels were consistently elevated to 20–22 mmol/l at peak level with a concurrent increase in plasma insulin levels to 2500–3000 pmol/l. Both plasma glucose and insulin returned to basal levels within 30 min after infusion of glucose was paused.

**Fig 1:**
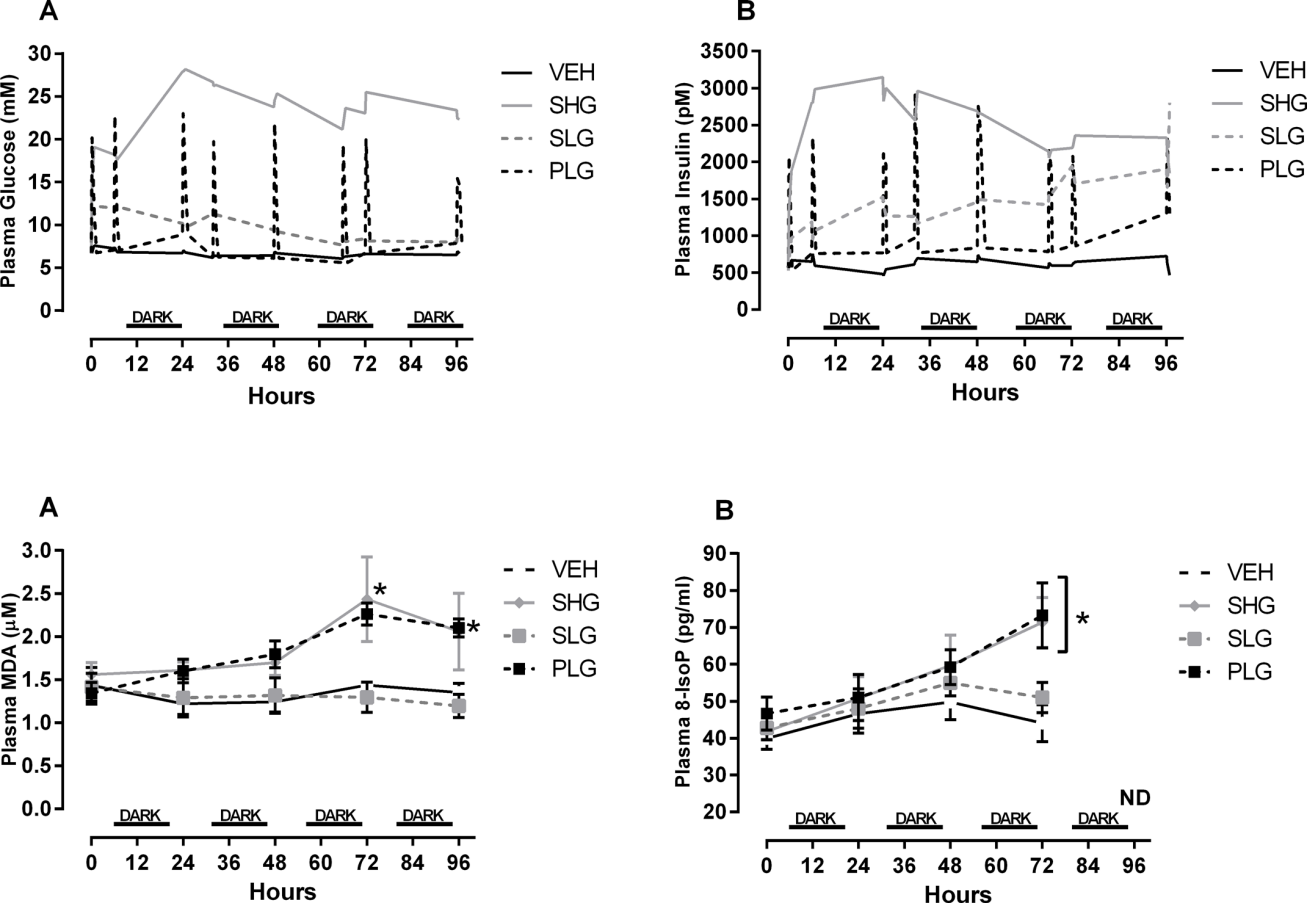
Pulsatile hyperglycaemia increases systemic oxidative stress status independent of glycemic exposure. Plasma glucose (A) and insulin (B) was followed throughout the complete study period. The PLG group were subjected to nine glucose pulses daily and the glucose levels were determined at selected time points covering the complete circadian rhythm. Plasma MDA (C) and 8-IsoP (D) was monitored daily at the exact same time point. Data are only means for plasma glucose and insulin and means ± SEM for plasma MDA and 8-IsoP, n = 7–8. *p < 0.05 vs. VEH.

Plasma MDA and 8-IsoP were measured as biomarkers of oxidative damage to lipids (Fig 1C & 1D). At baseline (T = 0 h) both plasma MDA and 8-IsoP did not differ among groups and during time course the Vehicle (VEH) and SLG showed no changes. For both plasma MDA and 8-IsoP ANCOVA revealed a significant difference in time x treatment interaction (p<0.05; both cases). In addition the post hoc test showed a significant increase in plasma MDA in the PLG group at 72 and 96 hours (p<0.05 vs. VEH; both cases), and in the SHG group at 72 hours (p<0.05 vs. VEH). Plasma 8-IsoP levels were also significantly increased in PLG and SHG at the 72 hour time point (p<0.05 vs. VEH; both cases).

### Liver Biochemistry

**Fig 2:**
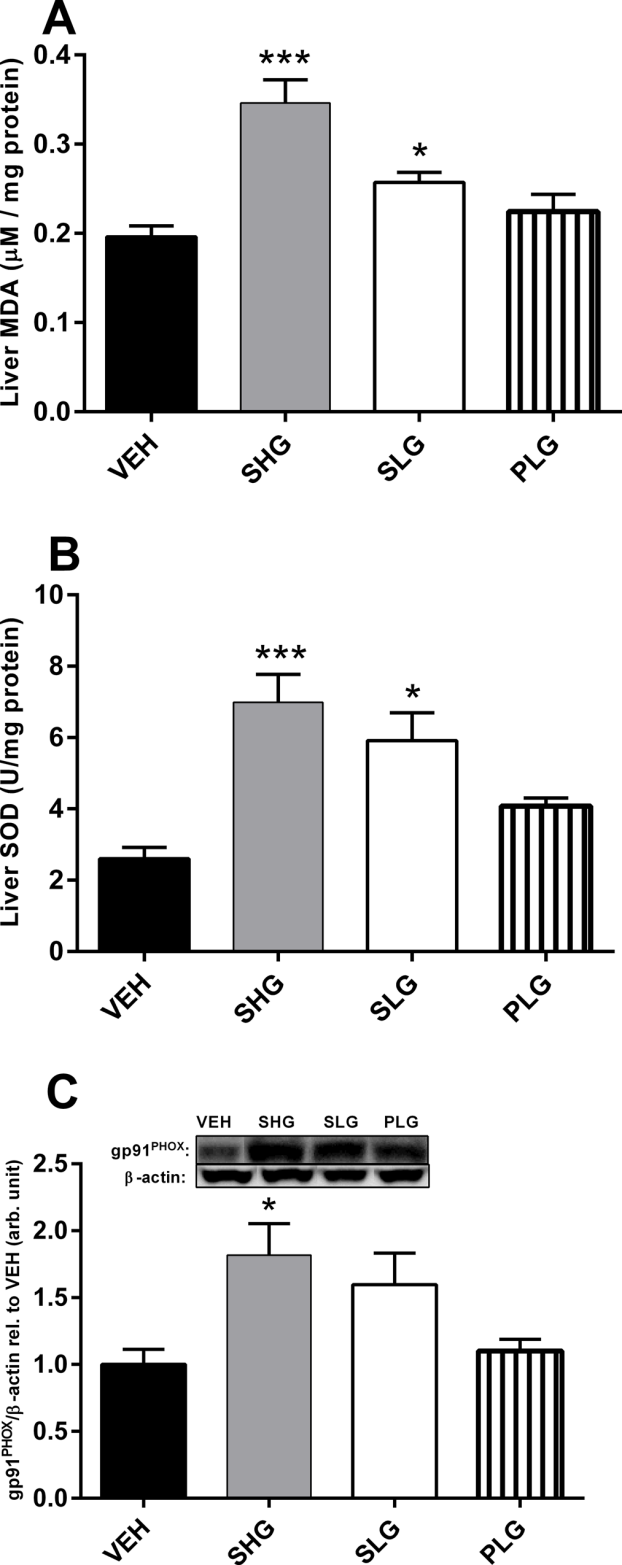
Sustained but not Pulsatile hyperglycaemia increases oxidative stress in liver. Liver MDA (A), SOD activity (B) and protein expression of gp91^PHOX^ (C) were determined in liver homogenates. Representive immunoblots of gp91^PHOX^ and β-actin are shown above the bars. Data are means ± SEM, n = 7–8. *p<0.05, ***p<0.0001 vs. VEH.

Overt hyperglycaemia (SHG) induced a significant higher level of liver MDA compared to other groups (Fig 2A; p<0.0001 vs. VEH; p<0.05 vs. SLG & PLG). Liver MDA was also significantly increased in the SLG group (p<0.05 vs. VEH). Similarly, we observed a significant increase in SOD activity (Fig 2B) in the SHG (p<0.001 vs. VEH) and SLG (p<0.05 vs. VEH) groups. Protein expression of gp-91^PHOX^ (Fig 2C), the major subunit of the NOX, was also significantly increased for the SHG group as compared to VEH and PLG group (p<0.05; both cases). Although not statistically significant (p = 0.067 vs. VEH), the gp-91^PHOX^ protein level was approximately 50% higher for the SLG as compared to the PLG and VEH group. Interestingly, neither of the analysed parameters were significantly affected in the PLG group.

### Liver Expression

**Fig 3:**
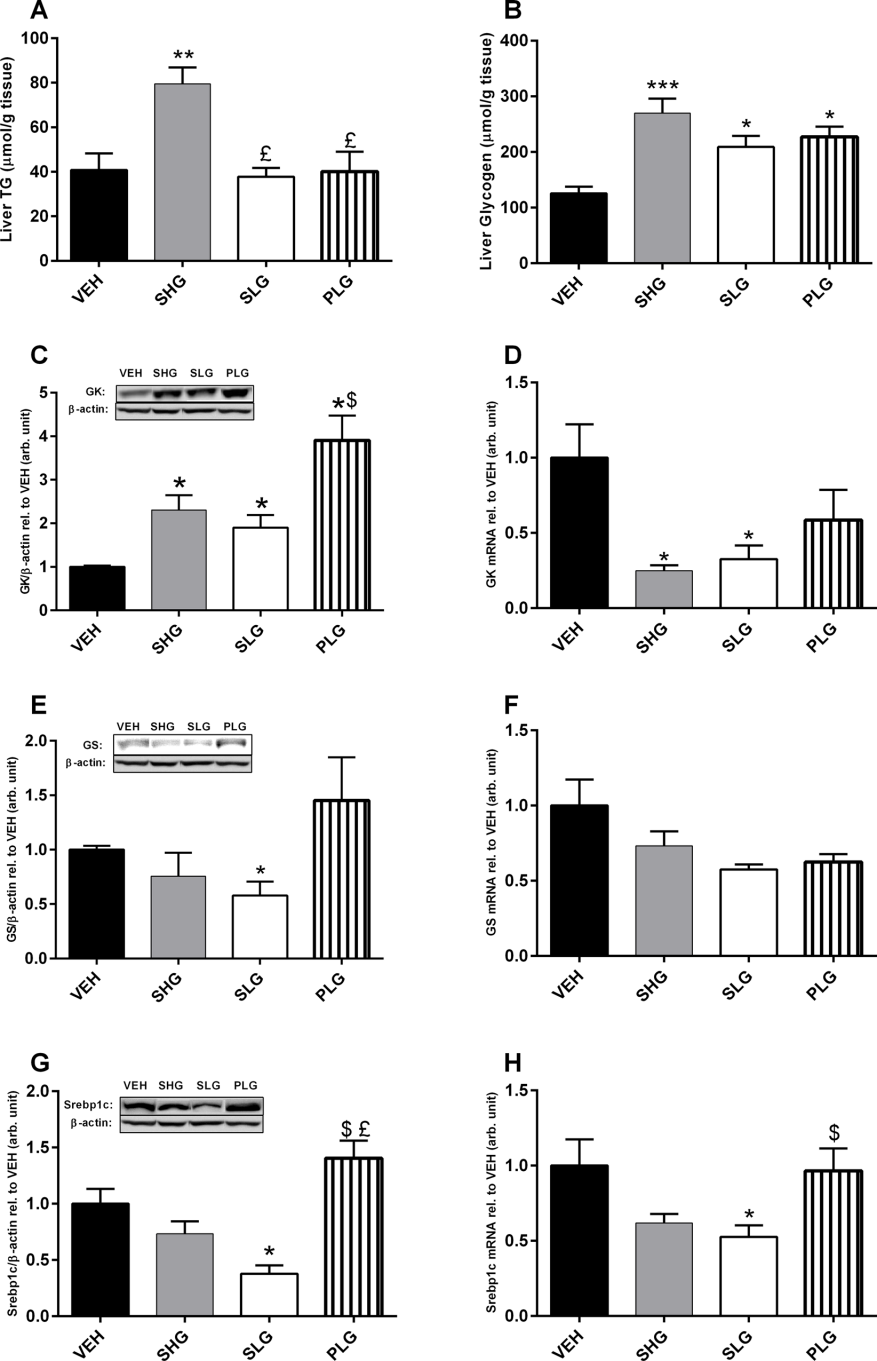
Chronic sustained hyperglycaemia down regulates key glycolytic enzymes in liver. After 96 hours of different glucose infusion paradigms liver was analysed for glycogen (A) and triglyceride (B) content and the expression level of GK (protein (C); mRNA (D)); GS (protein (E); mRNA (F)); Screbp1c (protein (G); mRNA (H)). Representive immunoblots of GK, GS, Screbp1c and β-actin are shown above the bars. Data are means ± SEM, n = 7–8. *p < 0.05, **p < 0.001 and ***p<0.0001 vs. VEH. $p < 0.05 vs. SLG. £p < 0.05 vs. SHG.

All intervention groups showed a significantly increased accumulation of TG and GLY in liver (Fig 3A & 3B). The SHG group showed a 2.5 fold increase in GLY and a 2 fold increase in TG levels as compared to the controls (p<0.0001; both cases). The PLG and SLG group showed moderate but significant changes in both GLY and TG (p<0.05; all cases). GK the rate limiting enzyme for glucose uptake in liver, was analysed by western blotting (Fig 3C). The SHG and SLG group showed approximately a 2 fold increase in GK protein level as compared to the controls (p<0.05; both cases). Interestingly, the PLG group had an even higher effect on the GK protein level, displaying a 4 fold increase compared to the controls (p<0.0001), which was also significantly higher than the SHG and SLG groups (p<0.05; both cases). In contrast, the GK mRNA level (Fig 3D) was significantly reduced in the SHG and SLG groups (p<0.05 vs. VEH; all cases) but not in the PLG group. Protein expression of GS (Fig 3E & 3F), the major regulator of glycogenolysis, was significantly reduced in the SLG group (p<0.05) but not in the SHG and PLG as compared to the controls. Finally, we assessed the hyperglycaemic effect on Srebp1c (Fig 3G & 3H). We observed a marked reduction in both protein and mRNA levels in the SLG group (p<0.05 vs VEH; both cases) but only at mRNA level for the SHG group (p<0.05 vs. VEH). The PLG group was not significantly affected as compared to controls.

### Aorta biochemistry

**Fig 4:**
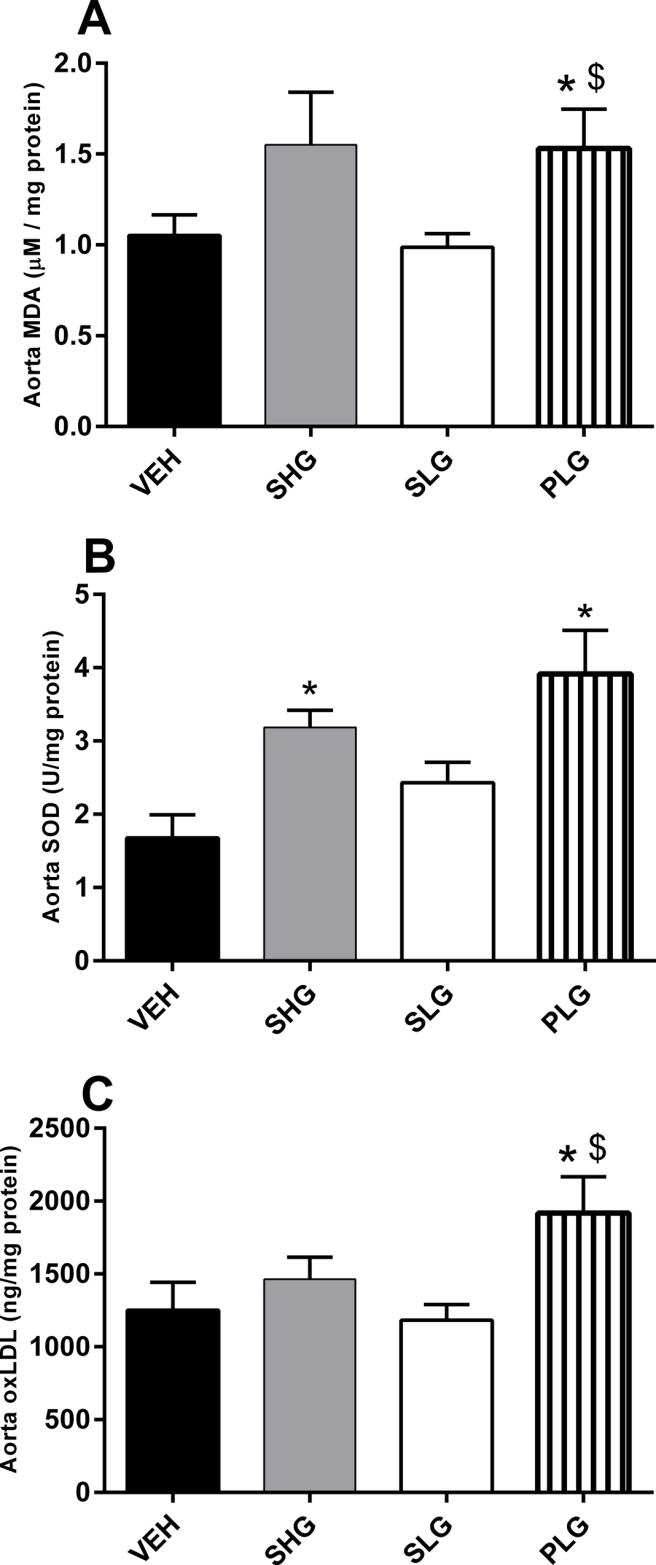
Pulsatile hyperglycaemia induced increased oxidative stress status and accumulation of oxLDL in aorta. Aorta homogenates were analysed for MDA content (A), SOD activity (B) and oxLDL (C) accumulation. Data are means ± SEM, n = 7–8. *p < 0.05 vs. VEH, $p < 0.05 vs. SLG.

**Fig 5:**
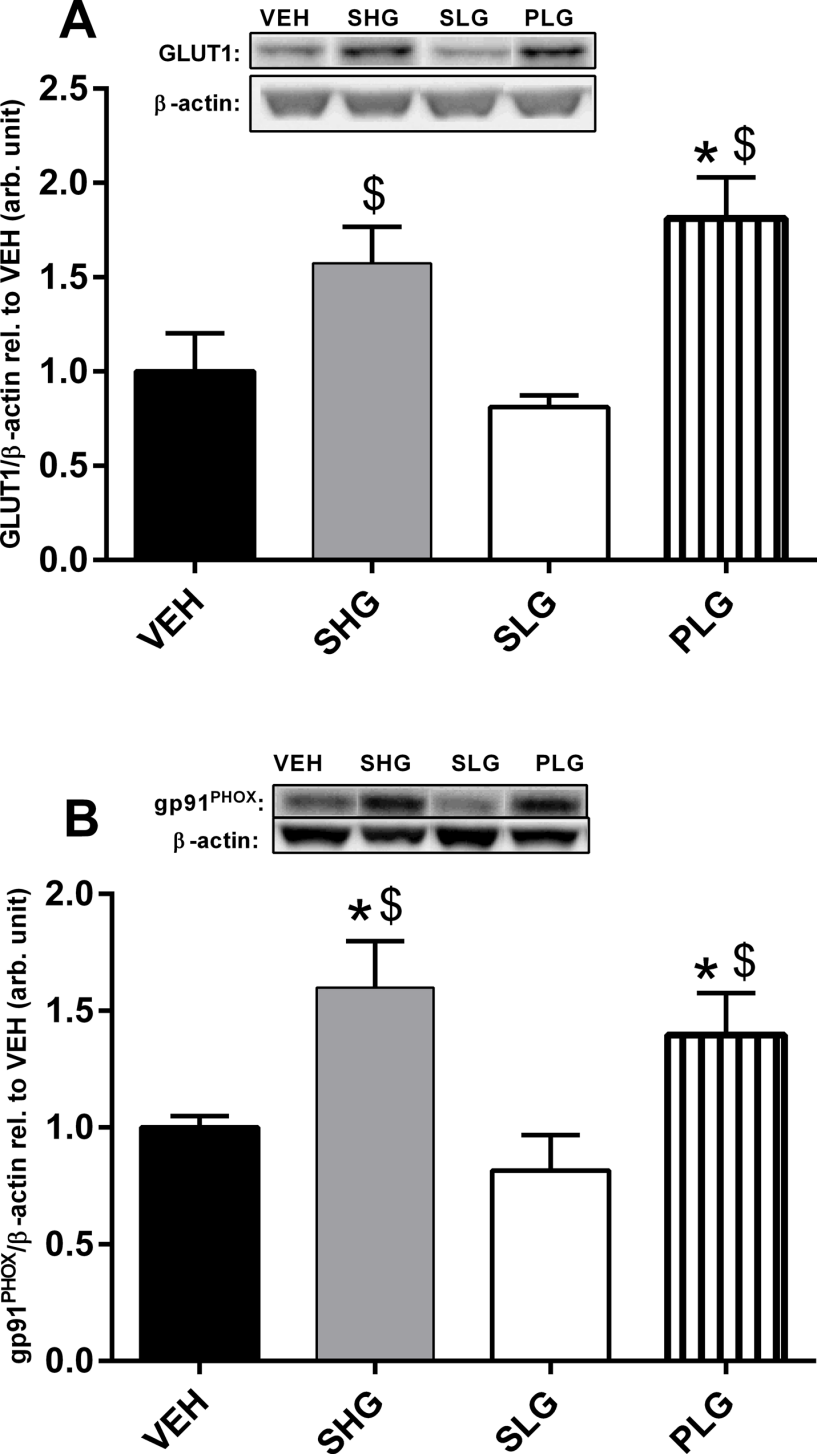
Pulsatile and chronic overt hyperglycaemia increased GLUT1 and gp91^PHOX^ protein expression in aorta. Western Blot analyses of GLUT1 (A) and gp91^PHOX^ (B) in aorta homogenates. Representive immunoblots of GLUT1, gp91^PHOX^ and β-actin are shown above the bars. Data are means ± SEM, n = 7–8. *p < 0.05 vs. VEH, $p < 0.05 vs. SLG.

Pulsatile hyperglycaemia induced significant higher levels of aortic MDA and oxLDL as compared to controls and the SLG group (p<0.05; both cases). The aortic SOD levels (Fig 4B) were significantly affected in the PLG and SHG groups (p<0.05 vs. VEH; both cases). In contrast to the liver, glucose uptake in aorta is not mediated by the action of insulin but primarily by facilitated diffusion through the GLUT1. In aorta, the PLG group showed a significant increase in GLUT1 and gp91^PHOX^ protein expression as compared to the VEH and SLG group (Fig 5, p<0.05; both cases). Furthermore, the abundance of GLUT1 and gp91^PHOX^ protein was also significantly increased in the SHG group (p<0.05 vs. VEH; both cases). Neither of the analysed parameters in aorta was affected in the SLG group.

## Discussion

Substantial efforts have been made to clarify if repeated incidents of short term hyperglycaemic “spikes” may be an independent risk factor for endothelial dysfunction and the development of macro vascular complications in diabetes[8,22,23]. Supporting this hypothesis recent reports both in humans and rodents have shown that intermittent hyperglycaemia induce higher levels of oxidative stress leading to cellular damage in particular in the cardiovascular system thus potentially resulting in diabetes related atherosclerosis and cardio vascular disease (CVD) [7];8;22;23). However, so far the exact sequences of biochemical events have not been elucidated.

In a previous study using lean rats with no signs of insulin resistance or dyslipidaemia, we showed that pulsatile compared to sustained hyperglycaemia may induce relatively higher levels of oxidative stress systemically but not at tissue level[24]. The lack of tissue effect may reflect that lean animals are metabolically extremely robust and to a large extent capable of handling hyperglycaemic episodes without experiencing severe induction of oxidative stress at tissue level.

Indeed, the development of diabetes related endothelial dysfunction is clearly composed of several metabolic disorders and besides hyperglycaemia, insulin resistance and dyslipidaemia have been shown to play important roles in endothelial function [25,26]. Insulin resistance has been suggested as one of the key components related to excess ROS production and oxidative stress[27]. Whole body glucose metabolism is composed of an “inter tissue” communication between insulin responsive and non-responsive tissues and paradoxically, down regulation of glucose uptake by insulin responsive tissues act as a protective counter regulation of excess glucose and thus leave VEC’s as important targets of hyperglycaemia mediated oxidative insult[14].

In present study, we added the challenge of a high fat diet to either sustained or pulsatile hyperglycaemia in rats with insulin resistance, dyslipidaemia (Table 2) and fatty liver for a more realistic model to evaluate the effects of hyperglycaemia on oxidative stress. We hypothesized that the liver, being the main regulator of glucose homeostasis, may exert important regulatory mechanisms of the glucose metabolism during different hyperglycaemic situations independent of the total glycaemic challenge. In fact, this regulation was hypothesized to affect the disposal and consequently the exposure of glucose to aorta and therefore have an indirect effect on the severity of oxidative stress e.g. on the vascular system in particular.

**Table 2:** Animal data. Mean ±SEM.

	DIO (n = 30)
Body weight (g)	679 ± 14
Plasma Insulin (pM)	640 ± 68
Plasma FFA (μM)	637 ± 44

MDA, is a widely used biomarker of cellular oxidative stress in diabetics[28], and studies both in diabetic subjects and in animal models have found increased lipid oxidation in both tissue and biological fluids[29,30]. A recent study showed a positive correlation between increased MDA in plasma and vascular dysfunction during hyperglycaemia both in insulin resistant and diabetic patients[31]. In this study, we also found increased plasma MDA levels both during overt sustained hyperglycaemia (PG > 20mM) and in animals with pulsatile plasma glucose profiles. ROS production has been shown to correlate with glycaemic exposure[18,32], but the observed effect on plasma MDA in our study seems to be independent of the total glycaemic exposure since the PLG group received one third of the amount glucose given to the SHG group. This suggests that a significant part of the effect on plasma MDA is independent of the total amount of glucose to which the rats were exposed to but rather dependent on the glycaemic profile. This effect on systemic oxidative stress was further supported by our findings on plasma 8-Iso-P also showing a significant increase when animals were challenged with pulsatile glucose profiles. Increased liver oxidative stress has been demonstrated both during chronic glucose infusion [33] and in insulin resistant animal models[34,35]. In support, we observed a marked increase in liver MDA and SOD in the groups receiving a sustained infusion of glucose but not in the PLG group. The membrane bound NOX subunit, gp-91^PHOX^, has been described as being the primary regulator of NOX and in studies where hepatocytes were subjected to chronic high glucose, expression of gp-91^PHOX^ was significantly increased along with increased NOX activity[36]. The significant increase in liver gp-91^PHOX^ protein expression for the sustained hyperglycaemic groups may indicate increased ROS production by the NOX and consequently, the induction of oxidative stress. This effect on oxidative stress markers was not present during pulsatile hyperglycaemia despite, being subjected to equal amounts of glucose as in SLG group. This may suggest that the liver is more prone to increased ROS production and oxidative stress when subjected to sustained hyperglycaemia but not during pulsatile hyperglycaemia. Studies have shown a direct link between higher glucose uptake rates and increased NOX. In order to answer these questions, we elucidated the effect on key enzymes in the liver responsible for glucose uptake and utilization. The GK protein levels were markedly increased during constant glucose infusion of glucose while the PLG group had an even higher protein level. However, at the mRNA level, only the chronically infused animals showed a marked reduction of liver GK while the PLG group was unaffected. The down regulation of GK expression in the sustained hyperglycaemic groups may indicate a lowering of net liver glucose uptake. However, it should be emphasized that this only represents a “snap shot” of the time course and multiple adaptive processes may have been involved during five days of glucose infusion. In fact studies have shown that the liver may exert biphasic controlling actions of glucose homeostasis in the hyperglycaemic state. At initial stages of hyperglycaemia, the liver facilitates glucose uptake while at later stages, the liver develop insulin resistance and facilitates glucose output[37,38]. The level of GK expression is primarily controlled by insulin and studies have shown that high fat feeding will induce liver insulin resistance with a concomitant reduction in GK expression and activity[39]. ROS molecules has been shown in several studies to be an important signal molecule and direct effects of increased ROS production in liver, muscle and adipose tissue has been demonstrated to consequently induce insulin resistance and act as a protective mechanism against further oxidative stress[9,10]. Assuming that this interpretation is correct, the liver plays an important role in protecting organs with insulin independent glucose uptake from excess glucose challenge only limited by its capacity to synthesize GLY and TG. However, this important metabolic regulation may be lacking or disturbed during pulsatile hyperglycemia. In order to address this question, we looked at oxidative stress status in aorta.

Cultured aortic endothelial cells subjected to intermittent hyperglycaemia has been shown to produce higher levels of superoxide as compared to cells subjected to sustained hyperglycaemia[5,6]. In agreement with this, we showed *in vivo* that pulsatile and to some extent also sustained hyperglycaemic status increased MDA and ox-LDL in aorta. Likewise, presumably as an adaptive preventive mechanism against increased ROS, the SOD activity was markedly increased as well. In this context, a reduction or a delayed adaptability of the antioxidant system to elevated ROS production may as well contribute to increased oxidative stress and favour the development of diabetes complications[40,41]. Thus a more detailed evaluation of the time dependency of the anti-oxidant response may explain some of the detrimental effects seen by pulsatile hyperglycemia.

Influx of glucose in endothelial cells is facilitated by GLUT 1 in an insulin independent manner[15] and excess glucose may activate PKC by several mechanisms, which consequently increases oxidative stress by activating NOX[16,18,42]. In connection we found a marked increase in GLUT1 and gp91^PHOX^ protein expression both during pulsatile and sustained hyperglycaemia. In diabetics, increased oxLDL accumulation in vessels has been shown to promote foam cell formation in vascular smooth muscle thus leading plaque formation and the development of atherosclerosis[19]. Additionally, oxLDL has itself been shown to produce oxidative stress in endothelial cells via activation of a NOX[43]. These findings may indicate that during pulsatile hyperglycaemia aorta may be subjected to excess glucose uptake affecting the activity of NOX thus leading to increased oxidative stress and ox-LDL accumulation. However the accumulation of oxLDL has been shown to be significantly affected by the LDL levels[44]. In our study we did not observe significant increases in plasma LDL levels for neither the SHG nor PLG group (S1 Table). A recent study in diabetics showed that even a tight control of LDL levels in diabetics was not sufficient to retain the increase of oxLDL levels during diabetes but was rather affected by the duration of diabetes [45]. It must as well be acknowledged that rats in contrary to humans lacks the enzyme, cholesteryl ester exchange protein[46], which may explain the very low levels of LDL but potentially also the lack of correlation between plasma LDL levels and the accumulation of oxLDL in our study.

In perspective, a role for insulin in this study must as well be considered. In a study by Ellger et al. where normal rats where chronically infused with either insulin or glucose or both, it was found that endothelial function was improved by insulin through its action of reducing hyperglycaemia[47]. In this regard, hyperinsulinemic conditions both in normal and T2D subjects and in rodents as well have been shown to increase hepatic de novo GLY and TG synthesis[48,49]. In support we observed a marked increase in liver GLY and TG in the SHG group. Insulin may exerts its effects by increasing the expression and activity of GLUT’s and lipogenic enzymes in the liver and thereby increase liver glucose uptake[49]. Furthermore, it has been shown that insulin may exert anti-inflammatory effects on the vascular system by regulating nitric oxide levels [50]. Additionally, studies have shown that insulin affects the levels of intercellular cell adhesion molecule-1, monocyte chemoattractant protein-1 expression and NFκB binding in human aortic endothelial cells in vitro [51,52] These actions by insulin, may hypothetically exert an indirect protection of the vascular endothelium during excess glucose exposure and hyperinsulinemic conditions as observed in the SHG group. In the PLG group, this effect of insulin may be absent due to the short term exposure to hyperinsulinemia. Thus in our studies, insulin may to some extent be a confounder and additional studies evaluating markers of oxidative stress and glucose disposal in insulin sensitive versus non-insulin sensitive tissues under hyperinsulinemic clamp conditions may be an approach in order to investigate these mechanistic consequences. Another limitation of our study is the lack of immune-histochemical measurements of tissue specific oxidative stress markers. DHE [53]and 3-nitrotyrosine [54]quantification in aorta tissue are often as measures of oxidative damage in the vascular endothelium. In this context, further studies in atherosclerotic prone animal models such as the apo lipo protein E knock out model [55] or low density lipoprotein receptor knock out model may be warranted to study the effects of glucose exposure and aortic plaque lesion formation[56].

Our study has a couple of limitations. First, As previously mentioned, we have used our study protocol with glucose infusion in normal rats with no signs of insulin resistance or obesity. In that study we did not observe any effects on oxidative stress at tissue level [24]. However due the natural variance in animal experiments having normal control without the insulin resistant phenotype in our study would have further strength the notion that insulin resistance might be an important accelerator of ROS production [27]. Secondly, our findings in liver and aorta are all at the terminal time-point. The “interplay” between organs in the regulation of glucose metabolism is considered to be dynamic and several compensatory mechanisms may be evident during a hyperglycaemic time-course[14,37,38]. Thus a time–course assessment of oxidative stress at tissue level would have been appropriate to include in the study in order to more precisely assess the regulation of glucose metabolism and the level ROS production at the individual organ.

In conclusion, we find that oxidative damage during different situations of hyperglycaemia may be highly compartmentalized where the cardiovascular system is primarily targeted by the pulsatile hyperglycemic profile whereas the liver is more disposed to oxidative damage by the chronic hyperglycemic profile. Thus our data provides substantial in vivo support for the hypothesis that pulsatile hyperglycaemia more potently than sustained hyperglycaemia induces oxidative stress in the cardiovascular system at a much lower glucose exposure. This is in agreement with the clinical evidence that pulsatile hyperglycaemia is an independent risk factor for diabetes related endothelial dysfunction, atherosclerosis and cardiovascular disease.

## Supporting Information

S1 Table Plasma TG, T-Chol, LDL and HDL were monitored daily at the exact same time point. Data are means ± SEM, n = 7–8. *p < 0.05; **p < 0.001 vs. VEH.
(DOCX)
