# Supplementary material for: Pulsatile Hyperglycaemia Induces Vascular Oxidative Stress and GLUT 1 Expression More Potently than Sustained Hyperglycaemia in Rats on High Fat Diet
Source: PLoS One. 2016 Jan 20;11(1):e0147412. doi: 10.1371/journal.pone.0147412 (PMC4720376; doi:10.1371/journal.pone.0147412)
Supplement: S1 Table — Data are means ± SEM, n = 7–8. *p < 0.05; **p < 0.001 vs. VEH. (DOCX) [file pone.0147412.s001.docx]

**S1 Table.** Plasma TG, T-Chol, LDL and HDL were monitored daily at the exact same time point. Data are means ± SEM, n=7-8. *p < 0.05; **p < 0.001 vs. VEH.

|  | Time (hours) | VEH | SHG | SLG | PLG |  |  | Time*Treatment | Time | Treatment |  |
| --- | --- | --- | --- | --- | --- | --- | --- | --- | --- | --- | --- |
| TG (mM) |  |  |  |  |  |  |  |  |  |  |  |
|  | 0 | 0.32 ± 0.03 | 0.40 ± 0.09 | 0.26 ± 0.06 | 0.42 ± 0.07 |  |  |  |  |  |  |
|  | 24 | 1.40 ± 0.24 | 0.52 ± 0.14 | 1.14 ± 0.42 | 1.57 ± 0.32 |  |  |  |  |  |  |
|  | 48 | 1.20 ± 0.15 | 1.58 ± 0.28 | 1.13 ± 0.37 | 1.09 ± 0.18 |  |  | <0.05 | <0.0001 | ns |  |
|  | 72 | 0.76 ± 0.15 | 1.70 ± 0.17 | 1.29 ± 0.16 | 1.60 ± 0.16 |  |  |  |  |  |  |
|  | 96 | 0.74 ± 0.09 | 1.52 ± 0.51 | 0.92 ± 0.22 | 0.94 ± 0.28 |  |  |  |  |  |  |
|  |  |  |  |  |  |  |  |  |  |  |  |
| T-Chol (mM) | 0 | 1.74 ± 0.18 | 2.15 ± 0.11 | 2.15 ± 0.13 | 2.05 ± 0.08 |  |  |  |  |  |  |
|  | 24 | 1.86 ± 0.13 | 1.12 ± 0.21 | 2.28 ± 0.41 | 1.82 ± 0.32 |  |  | <0.05 | <0.0001 | <0.01 |  |
|  | 48 | 2.50 ± 0.22 | 1.52 ± 0.10* | 2.37 ± 0.37 | 2.43 ± 0.13 |  |  |  |  |  |  |
|  | 72 | 2.64 ± 0.13 | 2.08 ± 0.21 | 2.20 ± 0.26 | 2.59 ± 0.09 |  |  |  |  |  |  |
|  | 96 | 2.53 ± 0.16 | 2.53 ± 0.26 | 2.14 ± 0.27 | 2.13 ± 0.12 |  |  |  |  |  |  |
|  |  |  |  |  |  |  |  |  |  |  |  |
| LDL (mM) | 0 | 0.45 ± 0.06 | 0.66 ± 0.06 | 0.59 ± 0.06 | 0.47 ± 0.08 |  |  |  |  |  |  |
|  | 24 | 0.31 ± 0.05 | 0.18 ± 0.03 | 0.39 ± 0.14 | 0.30 ± 0.03 |  |  |  |  |  |  |
|  | 48 | 0.54 ± 0.05 | 0.33 ± 0.16 | 0.50 ± 0.08 | 0.43 ± 0.04 |  |  | <0.01 | <0.01 | <0.05 |  |
|  | 72 | 0.68 ± 0.04 | 0.19 ± 0.02** | 0.36 ± 0.08* | 0.45 ± 0.05 |  |  |  |  |  |  |
|  | 96 | 0.61 ± 0.11 | 0.47 ± 0.09 | 0.29 ± 0.07* | 0.44 ± 0.04 |  |  |  |  |  |  |
|  |  |  |  |  |  |  |  |  |  |  |  |
| HDL (mM) | 0 | 1.30 ± 0.07 | 1.34 ± 0.09 | 1.50 ± 0.06 | 1.19 ± 0.17 |  |  |  |  |  |  |
|  | 24 | 1.25 ± 0.06 | 0.91 ± 0.09 | 1.21 ± 0.22 | 1.04 ± 0.18 |  |  |  |  |  |  |
|  | 48 | 1.50 ± 0.17 | 0.80 ± 0.15 | 1.29 ± 0.24 | 1.17 ± 0.11 |  |  | ns | ns | <0.0001 |  |
|  | 72 | 1.54 ± 0.09 | 0.80 ± 0.19 | 0.94 ± 0.18 | 1.28 ± 0.08 |  |  |  |  |  |  |
|  | 96 | 1.80 ± 0.11 | 1.01 ± 0.22 | 1.03 ± 0.23 | 1.55 ± 0.09 |  |  |  |  |  |  |

**Plasma Lipoprotein & TG profiles during hyperglycaemic challenge (Table 1)**

Plasma LDL levels showed a significant interaction between time and treatment (p<0.001). Interestingly the post hoc analyses revealed a significant difference for the SHG at 72 hours (p<0.001 vs. VEH) and for the SLG group at 72 and 96 hours (p<0.05 vs. VEH; both cases) but no changes in the PLG group. The HDL levels were significantly affected among groups (p<0.0001). In addition the post hoc test showed a significant reduction in the HDL levels for the SHG group (p<0.0001 vs. VEH). Reflecting the changes in plasma LDL and HDL the plasma T-Chol showed a significant interaction between time and treatment (p<0.05). The post hoc analyses showed a significant reduction in plasma T-Chol for the SHG at 48 h (p<0.05 vs. VEH). The plasma TG levels showed an increase for all groups during the time course but no changes among groups. Plasma glucagon levels were also measured (data not shown). At baseline all groups had similar plasma glucagon levels (5-7 pM). The controls did not show any changes in plasma glucagon levels during the time course whereas as expected due to the glucose challenge all the intervention groups showed plasma glucagon levels below detection limit (< 2pM).
